# 2. How is the economic assessment of vaccines performed today?

**DOI:** 10.1080/20016689.2017.1335163

**Published:** 2017-08-31

**Authors:** Baudouin Standaert, Rino Rappuoli

**Affiliations:** a Health Economics, GSK, Wavre, Belgium; b Research & Development GSK, Siena, Italy

**Keywords:** Budget, economic evaluation, cost effectiveness analysis, societal perspective, vaccines, value assessment

## Abstract

This paper describes how the economic assessment of vaccines is performed today. It discusses why it may be incomplete and explores potential approaches to adjust the analysis to be more comprehensive. Besides helping protect against serious disease, vaccines also help avoid mild disease episodes that may not receive medical attention but which have important societal consequences. They also benefit unvaccinated individuals by reducing disease transmission. Wider societal benefits may extend beyond a decrease in disease incidence, as lower transmission rates reduce the risk of epidemics, which in turn reduces the pressure on healthcare providers, and may improve the quality of care for patients with unrelated diseases. Vaccines also lower the use of antibiotics leading to less pressure on anti-microbial resistance. Conventional ICUA focuses on individual health benefits, like increased survival. Therefore, this approach may not adequately capture the wider vaccination benefits. We discuss differences between treatment and vaccine prevention in the economic assessment, and how ICUA has been adapted to cope with the inconsistencies. Although such adaptations may fulfil the demand of one specific stakeholder, they may not meet the needs of other stakeholders who operate at the societal level, such as ministries other than healthcare, employers, caregivers, and insurers.

## Introduction

The previous paper in this series identified challenges for assessing the total economic value of a new vaccine entering the healthcare market [1]. Four factors have the largest influence on measuring its value performance for the economic assessment [2]. First, the vaccine should preferentially be evaluated at the population level instead of the subject level. Second, the evaluation should be societal rather than individual, as vaccination has broad social impacts that do not apply to treatment. Thirdly, budget impact should primarily be considered, as the available budget is more critical than the cost-effectiveness result in decisions about financing the vaccine. Finally, the period of evaluation should be well defined linked to the objective whether disease-control, disease elimination, and/or disease eradication are to become the focus of the vaccination programme [3].

This paper discusses challenges in the current health economic assessment of vaccines [4,5]. Some simple hypothetical examples illustrate the differences between treatment and vaccine prevention, and show the limitations of the current incremental cost-utility analysis (ICUA) evaluations when applied to vaccination. Finally, we go through the adjustments made in the health economic assessments to address some of the issues identified [6–8]. The discussion considers whether these adjustments are enough to capture the full economic value of vaccines.

## Organisation of treatment versus prevention

A major benefit of successful vaccination is that something harmful – an infection with its consequences – does *not* happen [1]. In other words, ideally nothing should happen when vaccinated. This raises the question of how to measure the gain if nothing happens. The benefit of disease prevention is not easy to measure and needs to be evaluated under specific conditions. As detailed in the first paper, the situation of treatment and cure is more familiar, and its organisation is relatively straightforward to understand [9]. The process mainly starts when patients have complaints. With a complaint a patient can enter the healthcare system to obtain a diagnosis based on symptoms measured by a physician. The doctor prescribes a treatment that, if successful, will improve the symptoms and can lead to cure. The physician has many treatment options, and selects the one with the highest chance of success. This system is workable and sustainable if there is a social security system in place that finances the process of diagnosis and treatment. Everything is focussed on the individual. The financial value of the health gain for the individual patient as a result of treatment has been estimated at a maximum cost of between 20,000 and 50,000 monetary units /effect gained or 54–137 monetary units for a perfectly healthy day [10]. All new treatment options that enter the current healthcare market and are available for a doctor to choose have been evaluated within that scheme of extra payment for extra health gain, to a maximum level. If the physician changes treatment to raise the chance of treatment success for the patient, the system does not fail in its delivery because the available options have been evaluated in the same way.

Prevention is quite different from treatment and cure, both in the starting point and in the measurement of success. First, prevention by vaccination is not targeted at people who are already sick, because by then it is too late to prevent illness. Instead, prevention aims to reach as many of the at-risk population as possible. So the action of the medical corps is in the opposing direction to treatment; active outreach until all subjects are covered, rather than waiting for patients to present. The organisational consequences and logistics of vaccine prevention are therefore highly different from curative care. Many countries have developed a special health care structure for vaccination of infants and children separate from normal care [11]. With high vaccine coverage, the benefit at the population level is higher than summing the individual gain per vaccinated subject because herd effect potentially reduces disease risk in the unvaccinated population. So, measuring vaccine success should occur at the population level rather than the individual level. However, calculating preventative successes is a challenge because with good prevention nothing should happen and measuring nothing over a long period of time is not very exciting. It can lead to more errors and a tendency to weaken the continuation of vaccination (see next section) [12].

Shifting from treatment and cure to prevention requires major changes to delivery of care that may be challenging to organise quickly and well. It is certainly not as easy as switching between treatment options. For instance, when rotavirus vaccine was introduced in the European market in 2006, it took time (years) to have the vaccination implemented because of lack of budget availability, organisational changes required to start a new vaccination program, and the need to convince many stakeholders of the hidden benefits of the vaccine. The introduction could have been better prepared, with better understanding of the data from the randomised clinical trial, and identifying specific gains such as the reduction in nosocomial infections that were not measured during the trial [13].

## Simple quality of life assessment

Treatment always comes late in the process of disease development, while prevention occurs earlier, before symptoms appear. As a consequence, the accumulated quality of life (QoL)-benefit in a cohort will always be larger with prevention than with treatment. As discussed in the first paper, the higher QoL-gain with prevention than treatment could potentially justify a higher cost for vaccine prevention if cost setting is conducted in the same way as for treatment.

**Figure 1.**
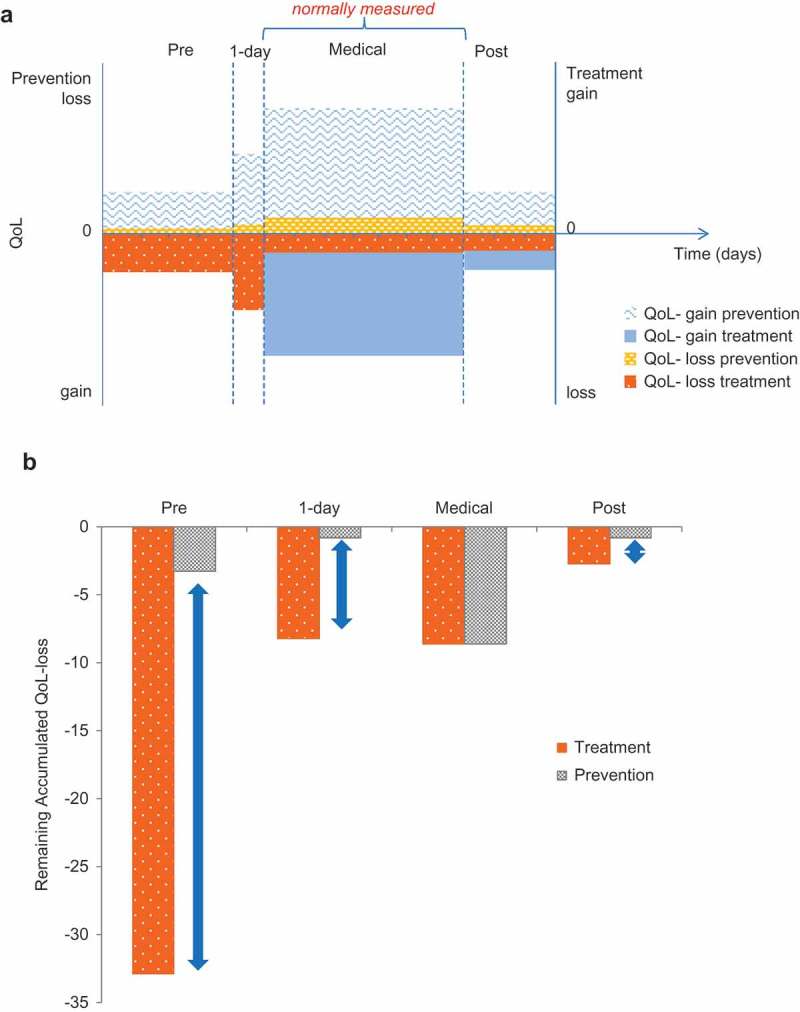
Comparison of treatment versus prevention: hypothetical example (a) Assessing the individual quality of life (QoL) gain or loss with prevention and treatment; (b) Impact difference between treatment and prevention at a population level (cohort of 100,000 subjects). QoL, quality of life

Table 1 illustrates the amount of difference in the QoL loss between treatment and vaccine prevention. It is a simple hypothetical disease case comparable to rotavirus and similar to other childhood infectious diseases [14] .The accumulated benefit gain expressed in reduced QoL loss is measured for a cohort of 100,000 subjects over a period of 1 year. In the cohort receiving vaccine prevention, the QoL loss due to the disease is only 13.56 QoL/year, almost 4 times smaller than the QoL loss in the cohort receiving treatment (52.47 QoL/year). That is because the vaccine prevents the QoL loss from the whole of a disease episode including the period of residual post-treatment impairment, with the QoL loss that does not receive medical care. The accumulated gain in less QoL-loss with a vaccine should therefore be higher than for a therapeutic intervention with similar efficacy. Figure 1 illustrates this visually.

**Table 1.** Overall and specific evaluation under medical attention only; and comparison of QoL-loss avoided for a cohort of 100,000 subjects by specific treatment versus vaccine prevention (hypothetical example).

				No specific treatment		Specific treatment		Vaccine prevention	
	Impact (Vaccine Efficacy)					0.9		0.9	
			QoL-loss	Days	QoL/year	Days	QoL/year	Days	QoL/year
**Overall evaluation**	Pre-medical	Disease	−0.1	4	−0.0011	4	−0.0011	0.4	−0.00011
	Medical	Disease 1st day	−0.2	1	−0.00055	1	−0.0005	0.1	−0.000055
		Disease subsequent days	−0.3	7	−0.00575	0.7	−0.0006	0.7	−0.0006
	Post-medical	Recovery	−0.1	3	−0.00082	1	−0.0003	0.3	−0.0001
	Total				−0.00822		−0.0025	−0.0007	
			Proportion^a^						
	Pre-medical	Disease	0.3		−32.87671		−32.877		−3.2877
	Medical	Disease 1st day	0.15		−8.21918		−8.2192		−0.8219
		Disease subsequent days	0.15		−86.30137		−8.6301		−8.6301
	Post-medical	Recovery	0.1		−8.21918		−2.7397		−0.8219
	Total				−135.6164		−52.466	−13.5616	
	**Gain in less QoL-loss**					**83.1506**		**122.0548**	
	**Additional gain in less QoL-loss**							**38.9041**	
			QoL-loss	Days	QoL/year	Days	QoL/year	Days	QoL/year
**Specific evaluation under medical attention only**	Pre-medical	Disease	0	0	0	0	0	0	0
	Medical	Disease 1st day	−0.2	1	−0.00055	1	−0.0005	0.1	−0.000055
		Disease subsequent days	−0.3	7	−0.00575	0.7	−0.0006	0.7	−0.0006
	Post-medical	Recovery	0	0	0	0	0	0	0
	Total				−0.0063		−0.0011	−0.00065	
			Proportion^a^						
	Pre-medical	Disease	0		0		0		0
	Medical	Disease 1st day	0.15		−8.21918		−8.2192		−0.8219
		Disease subsequent days	0.15		−86.30137		−8.6301		−8.6301
	Post-medical	Recovery	0		0		0		0
	Total				−94.52055	−16.849			−9.452
	**Gain in less QoL-loss**					**77.671**		**85.0686**	
	**Additional gain in less QoL-loss**							**7.3973**	

^a^Proportion of the total population cohort.

QoL, quality of life.

The gain in less QoL-loss from vaccine prevention extends over a longer period and a larger total area (i.e., larger total gain) than the gain seen from treatment (Figure 1a+ b). The additional gain in less QoL-loss with vaccine prevention is 38.9 units in the cohort, 32% higher than for treatment (Table 1). The part of the disease burden that would not be affected by medical treatment is prevented by the vaccine. In the absence of data, we often only report the QoL-gain measured during the medical observation period which tends to be very low (7.4 units instead of 38.9 [see Table 1]). It should be noted that in the example presented here the main additional benefit of preventative vaccination is focussed on disease events avoided during the pre-medical attention period. However, some infectious diseases cause their bulk of QoL burden during post-medical attention, such as meningitis, that isn’t as large in frequency but has a more severe and long-lasting disease burden preventable by vaccination.

## Consequences at individual and population level

Although prevention targets a greater fraction of the disease burden than a therapeutic intervention, the individual recipient may not be aware of any immediate personal benefit because they are not ill when vaccinated and the health gain occurs in future disease episodes avoided. That sort of benefit is challenging to measure, especially when there is a long interval between the initial vaccine administration and the health benefit, e.g., vaccination of adolescent girls against human papillomavirus (HPV) to prevent cervical cancer in adulthood [15]. Therefore any new vaccine needs close monitoring to detect events not prevented by the vaccine [16].

The absence of immediate personal benefit to the recipient may have adverse consequences. There may be a tendency to weaken the continuation of the vaccine programme, leading to lower coverage and an increase in susceptible individuals at risk of infection [17]. Decreases in disease incidence with successful prevention may even lead to errors such as more false-positive screening test results in cervical-cancer smears [18]. Being exposed to such specific problems, it highlighted two critical points to be considered. One is to collect as much real world evidence data when new vaccination programmes are initiated and related to that is the importance to remain most vigilant about safety concerns of any new intervention introduced in a healthy population.

Vaccine impact is normally measured by comparing the vaccinated population against historical data or a separate unvaccinated population, usually in terms of reduction in disease events [19]. In contrast, vaccine effectiveness measurement needs a case-control evaluation design that is usually more complex to organise and to calculate [20]. The benefit at population level may be very large, and at the extreme the disease may be completely eliminated. However this cannot easily be quantified at the individual level over a precise period of time. It must be measured within a population for reporting meaningful results.

As detailed in the first paper, vaccination differs from both therapeutic interventions and other forms of preventive interventions (such as taking statins to reduce the risk of cardiovascular disease) because transmissible diseases affect people in the wider population.

A consequence of high vaccine coverage is that the population receiving vaccination is very different from the population receiving a therapeutic intervention. Vaccination targets the whole at-risk group, whereas the therapeutic intervention is limited to people who already have the disease and have sought medical treatment. Although the health benefit per disease episode avoided is larger with vaccination than with treatment (Figure 1, Table 1), the benefit per person may be lower with vaccination because it is diluted among the whole at-risk population, not all of whom would have experienced a disease episode that would be avoided by the vaccine. The degree of dilution depends on the size of the population targeted for vaccination and the disease incidence. It is offset by the benefits to the unvaccinated population such as the herd protection.

**Figure 2.**
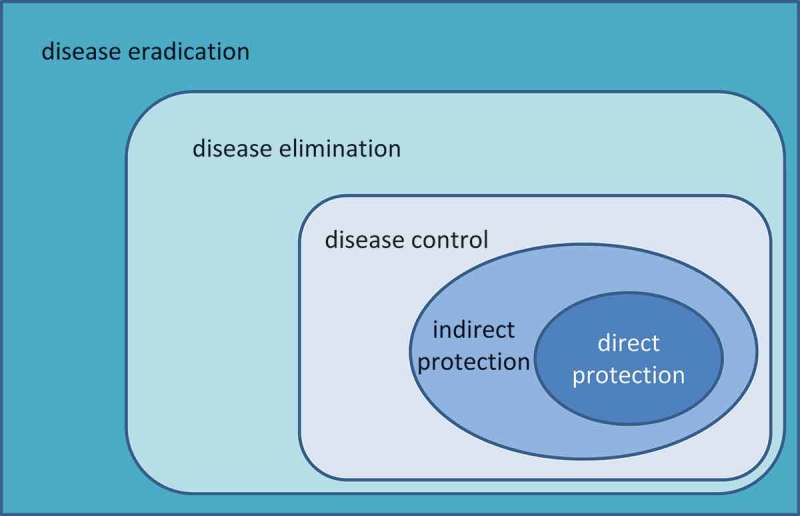
Schematic view of the different levels of vaccine benefit.

The total population benefit of vaccination may be thought of as a series of expanding concentric circles (Figure 2) [21], fulfilling different health objectives over its introduction in a population. First, vaccinated individuals are prevented from catching the disease (*direct protection*). Second, unvaccinated individuals in the immediate environment of vaccinated individuals are protected because disease transmission is reduced (*indirect or herd protection*). Third, if vaccination coverage is maintained over time it can reduce the occurrence of disease outbreaks or epidemics in the community (*disease control*). Fourth, if coverage is high enough for long enough it may be possible to eliminate the disease entirely from a region (*disease elimination*), and fifth if all regions of the world are able to eliminate the disease and the pathogen it reaches *disease eradication* status. This would achieve permanent protection and the vaccination programme can be stopped, although this is rare in practice (since it is possible only for pathogens having only a human host). Effective disease control leading to eradication may allow large-scale societal shifts. For example, healthcare providers could allocate resources to treat other diseases or to enhance quality of care (QoC), and governments could fund other health or social priorities [22].

One of the main issues, from the broader picture of the impact of vaccine beyond the direct and indirect effect protection, is to correctly evaluate the benefit of disease control over disease eradication expressed in monetary terms. Since this benefit is difficult to assess, it is often ignored. Meanwhile, measurement by contrast is one way to go on this evaluation: what happens if treatment fails? This evaluation way of preventative benefits has been poorly explored so far but could be developed with the case of antibiotic resistance which is steadily on the increase [23].

## Simple economic assessment

**Figure 3.**
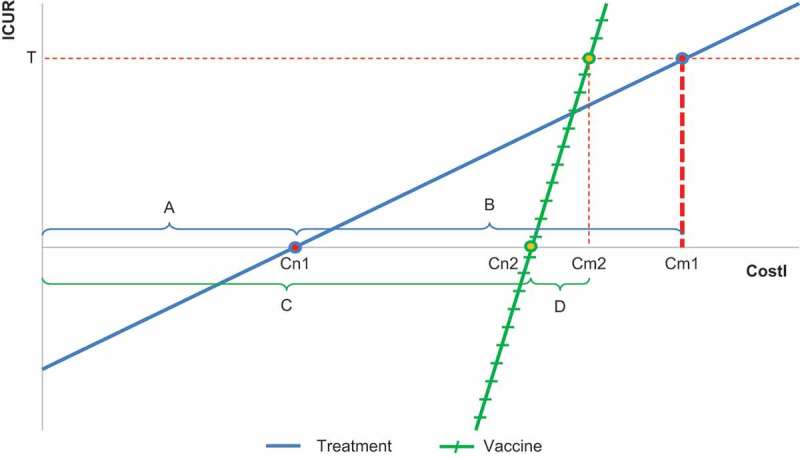
Incremental cost-utility ratio (ICUR) per QALY gained versus intervention cost (CostI) comparing treatment (blue line) versus vaccine (green line). ICUR, incremental cost-utility ratio; CostI, intervention cost; QALY, quality-adjusted life-years. (A) Cost-offset when two treatments are compared; (B) The difference in cost between the cost-neutral point (Cn1) and the cost where the line reaches the threshold (Cm1) for a treatment compared with another treatment; (C) Cost-offset when a vaccine is compared with a treatment; (D) The difference in cost between Cn2 and the cost where the line reaches the threshold (Cm2) for a vaccine compared with a treatment; T, threshold value.

Vaccination thus has a broad societal and public health impact that is best understood at a population rather than at an individual level. Of the five vaccination benefits in Figure 2, only the first (direct protection) relates to vaccinated individuals. All the others relate to the wider population, and the last (disease eradication) also relates to future generations. Therefore, a full assessment of the value of a vaccination programme should include community benefits. Current ICUA techniques were developed for measuring the effects of treating individuals, and may omit societal benefits that cannot easily be measured in quality-adjusted life-years (QALYs). Thus, the incremental cost per QALY gained may behave very differently when used to compare a vaccine with a treatment than when used to compare two treatments. This is illustrated in Figure 3.

The relationship between the incremental cost-utility ratio (ICUR) and the cost of a new intervention (CostI) can be expressed as a linear function (y = ax + b), where y (ICUR) is the dependent variable and x (CostI) is the independent variable. Additional equations and variables help to identify which parameters define the slope of the line (a) and the intercept (b). The cost-neutral point (Cn) is the cost at which the ICUR is zero (because there is no cost difference between the new intervention and the comparator at that point). The maximum cost (Cm) at which a new product is still cost-effective is the cost at which the line intersects the threshold value (T). The cost range over which the intervention is cost-effective is the cost over which its ICUR lies below the threshold value (T).

When two treatments are compared (blue line in Figure 3) the cost-offset (A) is likely to be marginal because the new treatment avoids only some of the costs of a disease episode, and so Cn1 could be low. When a vaccine is compared with a treatment (green line in Figure 3) the cost-offset (C) will be larger because all the treatment costs of disease episodes could be avoided, and therefore Cn2 can be high. Conversely, the QALY benefit from vaccination is diluted among a larger population than the QALY benefit from treatment, and thus the slope of the line is steeper. As a result, the difference in cost between Cn and the cost where the line reaches the threshold (Cm) is smaller for a vaccine compared with a treatment (D) than for a treatment compared with another treatment (B). This is what is found in a developed country. The reasoning is however different in a developing country (see below) [24].

The focus of ICUA has always been on determining the B and D ranges for any comparison undertaken, because those ranges justify the cost increase for the QALYs gained under the threshold T. Cost ranges A and C were less of a concern, because A will be marginal, and neither are directly linked to the threshold T that plays a perverse role in setting the Cm regardless of the cost-offset obtained. ICURs below the Cn are conventionally described as ‘dominant’ or cost-saving and those ICUR values may not be further quantified because of difficulties in interpreting the results.

Conventional ICUA is appropriate for the evaluation of therapeutic interventions providing benefits that can all be expressed in QALY gains at the individual level. The relationship between ICUR and cost provides useful information over a broad cost range (B in Figure 3). However, problems may arise when practitioners trained in these techniques make the natural attempt to apply the same approaches to vaccines. The wider benefits of vaccines such as herd protection, prevention of mild (non-medically attended) disease, and reduced pressure on healthcare providers during epidemics with potentially better QoC for other patients, are not adequately included in this economic analysis. Failure to incorporate such benefits distorts the analysis. A better assessment of range C is needed, where most of the gain of a vaccine is present. This will help to report more appropriate vaccine costs than in current analysis [25].

A major difference between economic evaluation of vaccines and therapeutic drugs is that the economics of therapeutic drugs are driven by the incremental benefit, whereas the economics of vaccines are driven by the cost-offset. With a therapeutic drug, a higher incremental benefit can justify a higher cost even if a threshold is defined for the ICUR. New therapies therefore focus on obtaining more benefit to support a higher cost.

With vaccines, the effect is felt across a population, and the effect per vaccinee decreases the more the target population is widened. In theory, if the target population could be very narrowly defined to include only individuals who benefit directly from vaccination (i.e., only those who would otherwise have gone on to contract the disease), the benefit per individual increases and the same logic as for drugs supplies. However, the major economic benefit generated by vaccines in developed countries is in avoiding costs, more than on the effect side. Effective prevention of disease episodes avoids hospitalisations, medical visits, lab tests, drugs, etc., and the cost-offset can be substantial. This is where the incremental cost-effectiveness analysis for vaccines may fail, because conventional thresholds are set for the QALYs gained but not for the level of cost-offset achieved. As cost-offsets could be marginal for drugs but large for vaccines, this is a critical difference. Vaccines could become attractive to a decision-maker if there is little extra cost related to the QALYs gained. Major cost-offsets may occur in parts of society other than healthcare because vaccines avoid many disease events that do not receive any medical attention but impact good societal functioning, affecting labour force availability and the leisure time of the population.

This becomes a problem in developing countries, where there is little investment in healthcare and consequently little potential for cost offset. The disease burden is so high that any new intervention introduced could result in a large benefit, theoretically resulting in a high cost that is cost-effective but not locally affordable.

Therefore, ICUA is not very helpful for decision-makers either in the developed or developing world. If it shows marginal benefit (developed world), the lower the benefit the more exponential the behaviour of the ratio, and the Cm that can be obtained is close to the Cm-offset. If the benefit is large (developing world), the price range at which the new intervention is cost-effective also becomes extremely large which is a little paradoxical for low income countries, and the cost-offset is likely to be close to zero because of the low healthcare investment. The decision-maker still does not know the real value/cost for the new intervention. Thus, neither the decision-maker nor the producer is really pleased with economic analysis using ICUA. The reality is that the decision-maker has often a limited budget, and the vaccine producer has a production cost and needs a profit margin to expand his business.

## Failures in using ICUA for vaccines

There are several reasons why conventional economic analysis may have difficulty in fully assessing vaccine benefits.

Some vaccination benefits are maintained over long periods, sometimes over a lifetime, or might only be obtained many years after being vaccinated. For example, the major benefit of HPV vaccination for adolescent girls is the prevention of cervical cancer in middle age, perhaps several decades later [26]. Clinical trials are quite impractical or unethical over such a long timescale, and therefore the economic evaluation of vaccine prevention at the beginning of a vaccination programme will rely mainly on modelling. While in theory it is possible to model vaccine effects over a lifetime, the models tend to be complex, are subject to a higher degree of uncertainty as the timescale extends, and can be highly sensitive to technical evaluation methods such as discount rates. In contrast, many therapeutic interventions provide symptom improvement over short time periods that can be directly observed in clinical trials, and modelling can use simple approaches such as decision trees [27]. Modelling is a critical concern in the economic assessment of vaccines at launch, and many techniques for model acceptance and calibration have been applied to increase the credibility of the results obtained with the assumptions made. Extensive sensitivity analysis is necessary to report results. It needs to be presented with credible boundaries for each variable in the model about which there is doubt of uncertainty in order to better understand the impact of some values on the end-results, separately (univariate) and/or combined (probabilistic sensitivity analysis).

The application of discounting rules to the long-term benefit of vaccine prevention is currently a topic of vigorous debate [28]. Positive discounting heavily decreases the projected accumulated benefit over time. But three questions animate the discussion: should we apply a different (lower) discount rate for effect than for cost and how different should it be; what is the appropriate discount rate for cost if the long-term interest rate on governmental bounds remain low due to low economic growth; the last question is more technical and related to the right approach of discounting in a population model that should be different from a cohort model. In addition another discussion point appeared recently: as future generations may benefit from prevention techniques applied today that save healthcare resource use in the future, a negative discount, as often applied now in environmental economic evaluation studies, may have to be considered and could be more appropriate [29].

Herd protection is a positive externality that benefits people in the population who do not receive the vaccine. The amount of herd protection depends on many variables such as the coverage rate, transmissibility of the disease, and population mixing between different groups [30,31]. These benefits are not easily included in conventional economic analysis. In theory, herd protection can be estimated using dynamic transmission models, but they are complex to construct, operate and explain, and they require detailed data that may not be readily available [31]. The difficulties in modelling herd protection correctly may often lead to its entire omission, especially when probabilistic sensitivity analysis is required. In addition, a big difference can be seen between what models predicted and the observation in real life as is the case for rotavirus vaccination in the UK [32]. A major reason for that is that not always the right pattern of infection processes are modelled, such as different sources of infections and the inadequate measurement of the vaccine coverage rate in the targeted groups. There may also be negative externalities such as age-shifts in infection that may occur if susceptible groups remain unvaccinated because of low vaccine coverage rates or because of vaccine waning processes. These can also be projected using dynamic models. They are sometimes observed in real life, but often adjustments are quickly made to avoid the predicted modelling conditions.

A third issue is to identify the most appropriate at-risk group to receive vaccination. This will define the total cost of the vaccine and the amount of dilution of the health benefit. It will depend on the amount society is willing to pay to reach a given disease risk in the population. This is typically a concern for catch-up programs and the selection of the target age-group for vaccination [33]. A combination of cost-effectiveness analysis and budget impact analysis can estimate the value for money and the feasibility of the programme, respectively.

Fourth, improved disease control and reduction in frequency and size of epidemics can interact with other healthcare programmes to benefit people in the population with unrelated diseases who share the same healthcare facilities. Reduced medical visits and hospital admissions during epidemics may allow hospital beds to be re-allocated to people with other diseases, for example people requiring elective surgery who might otherwise be crowded out during an epidemic. In addition, avoiding overcrowding and the staff pressure that can result from a spike in admissions during an epidemic may decrease nosocomial infections and improve the QoC [34]. Conventional health economic analysis has no precise method to include such a benefit, and it is not usually included in economic assessments. One way to estimate the QoC-improvement in monetary terms is to simulate the amount of extra-hospital investment needed to reach the same level of QoC obtained with the vaccine introduction using optimisation techniques [35].

Fifth, vaccine prevention acts earlier in the disease process than treatment and avoids mild disease events that do not receive medical attention. The number of such episodes may be large [36], especially during epidemics. Their impact may be significant to patients, families, employers and insurers, if people have to take several days away from normal activities to recover or to care for a sick family member. Mild disease is not included in economic assessments from the perspective of the healthcare system because it does not affect healthcare resource use. In theory, mild disease could be included in health economic assessments by estimating the number of episodes and the average economic impact (e.g., number of days off work). However, in practice the required data are often lacking because the frequency of mild disease is commonly hidden in reported estimates of the disease burden, as it receives no medical attention [37]. Some data sources unrelated to disease management may help in identifying absenteeism at work that could be an indirect indicator of mild disease burden [37].

Sixth, as well as avoiding early disease stages, vaccination also avoids periods of post-medical recovery and rehabilitation after illness. Table 1 and Figure 1 illustrate this for a hypothetical childhood disease where a high disease burden is noted pre-medical attention, but other conditions are possible related to a high bulk post-medical attention such as for meningitis. This period of recovery is especially important amongst elderly people, who may have very prolonged or incomplete recovery after hospitalisation for influenza, for instance [38]. The benefits of vaccination in avoiding such long-term effects could be large, although the increased recovery time has not yet been well quantified and is not usually included in economic assessments [39].

Seventh, vaccine prevention of infectious diseases has intangible benefits that cannot easily be measured in monetary terms, such as avoiding pain, discomfort, unpleasantness or disruption due to the disease or cumbersome interventions [40]. For example, HPV vaccination requires a simple injection that could avoid the need for future investigation and treatment of pre-cancerous abnormalities and/or cervical cancer, and may be able to reduce the frequency of Pap smears for screening. It could also improve fertility rates by avoiding conisations to remove advanced pre-cancer lesions or hysterectomies undertaken to avoid cancer in the absence of vaccination. Respondents expressed a strong willingness to pay for a HPV vaccination programme in a discrete choice experiment [41]. Such benefits may be under-reported, or may not be transparently evaluated. They may be easier to include in evaluations if they can be expressed in monetary terms such as productivity gains. These are essentially societal gains and will be maximised by greater vaccine coverage, although they may also be part of individual benefit. To facilitate their assessment it has recently been proposed to categorise them into three groups [40]:

- outcome-related productivity gains such as improved cognition and physical strength after full vaccination during childhood;
- behaviour-related productivity gains such as improved fertility after the introduction of HPV vaccination;
- community externalities such as prevention of antibiotic resistance with the introduction of pneumococcal conjugate vaccines [40].

Eighth, in some situations diseases prevented can have effects that should be considered beneficial for other stakeholders than those evaluating the healthcare programs. For example, Ministries of Finance would welcome keeping the population as healthy as possible throughout life. Under such circumstances the population becomes full consistent tax-payers which may benefit the government to improve their economy. This may lead to potential macroeconomic benefit as well. Special fiscal modelling on rotavirus disease has been developed to demonstrate the potential benefit at fiscal level of avoiding deaths during childhood growing up to become additional taxpayers [42]. As another example, malaria-endemic countries have recorded lower economic growth than countries without malaria over extended periods [43]. Possible ways in which malaria could inhibit economic growth include restricting flows of trade and investment, exposing people to sudden catastrophic costs that eat into their savings and prevent them investing, and reducing the productive capacity of the population due to long-term disability [43]. Effective disease prevention could have beneficial macroeconomic effects in such situations, but this is not included in current economic evaluations.

Finally, the cost-effectiveness of a vaccine could be very different depending on whether the disease is under control. A major shift in economic value can be expected when the disease becomes endemic and under control, compared with when it is epidemic with sporadic outbreaks. In the latter condition, the inherent value of a new vaccine may be critically dependent on the interventions societies wish to fund to improve social welfare and to move to higher control levels [44]. The process of an economic evaluation of a vaccine could therefore be very dynamic with clear changes in the results if the focus is on disease reduction, control or elimination but that is more part of a political strategic direction than on model results only.

These are examples of the many domains where vaccine prevention can provide benefits that cannot be assessed in the same way as treatment interventions. A new framework for the economic analysis of prevention may be needed to broaden perspective and to place prevention in the context of a health economic value programme that provides social welfare benefits as well as individual benefits [2].

## Attempts to recover the full economic value of vaccines

Table 2 summarises some of the attempts that have been made to adjust the ICUA method to correct for the issues discussed. The ICUA approach remains the main focus, perhaps reflecting the demand of stakeholders familiar with this type of analysis. However, as vaccine impact occurs mainly at the societal level, many other stakeholders could be interested in an economic assessment of vaccination. Their evaluation may differ from the conventional health economic view, because of differences in focus (health gain versus financial safety, for instance), perspective (healthcare versus government), or the starting point of evaluation (fixed versus flexible budget). This may necessitate approaches other than ICUA to economic assessment. It is therefore worth considering other methods of economic assessment that tackle the problem from a different angle, if available. This may help strengthen the position of the new product in a broader environment.

**Table 2.** Evaluation tools for assessing the economic value of vaccines.

Name	Benefit	Cost	Summary	Value	References
Cost-effectiveness using cost-utility gain	Expressed as QALY gained	All different cost items to be considered	Ratio of the cost-difference divided by a QALY difference being under or equivalent to a local threshold	Incremental cost-effectiveness result expressed in money terms	[45]
ECEA	Expressed in QALYs gained + financial risk protection (reduction in out of pocket payment)	All different cost items to be considered	Cost effectiveness ratio for the health gain and % of financial risk protection achieved	Combines economic and social indicators to prioritise new interventions at household level	[46]
Cost-Benefit	All benefits are expressed in monetary terms	All different cost items to be considered	Select the intervention with the highest cost value for society	Every benefit is evaluated in monetary terms and equal money weight is given to each item in the equation	[25,47]
Cost-Consequence	Selects an item of importance to be improved with the new intervention	Medical and non-medical cost	Example is the payment made per hospitalisation avoided, or medical visit avoided	No value is given to events that have been avoided.	[48]
Portfolio management	Specific health goal to optimise through the use of different vaccines in a specific sequence over time	Budget constraint in the analysis, but other constraints can be included as well such as logistics	List of vaccine introduction in a multi-year, multi-budget plan with the highest health outcome benefit to be achieved within a specific time frame	Answers questions on the order in which vaccines should be implemented and what budget plan guarantees the highest health benefit within a specific time frame	[49]
Optimisation modelling	Evaluates a single vaccine introduction in terms of reaching a certain health goal within a time and budget frame	Budget limitation will identify how to reach the health goal	Optimisation is different from cost-effectiveness. Optimisation makes the link between budget and health goals to be reached within a time frame	Many decision makers prefer optimisation analysis above cost-effectiveness analysis because it uses the normal budget framework and it allows better combination of options	[24,50,51]
Return on investment	What is the return in tax payment if the population is kept healthy through vaccination?	Investment in vaccination, cost-offsets, extra payment in education, pension	Ministry of Plans and Finance are comfortable with the approach because of the terminology used (IRR)	Open the dialogue with decision-makers other than Ministry of Health	[42,52]
Poverty trap avoidance	Proportion of households staying above the income threshold of poverty	Household spending per time unit	The new intervention avoids sick conditions in households which improves productivity and reduces cost for medical care which avoids the fall into the poverty trap without openings of recovery	If more households stay away from the poverty trap, it may improve the local economic and welfare condition of the community under study	[53–55]
RSA	Mitigating financial risk with access to vaccines; cost-based deals instead of performance based	Payer’s budget is at risk with high investment at start	Financial risk through uncertainty in effectiveness, safety, uptake, supply, real-world implementation	Assessment through dose schemes, target population, herd protection and delayed benefit	[56]
Macro-economic assessment	Impact of a vaccination program on the improvement of macro-economic indicators such as GDP	GDP per capita and vaccine cost	Extended control of infectious diseases in children with vaccination programmes may impact the whole economy of a country	Not many health interventions are able to demonstrate a real impact on the economy through better specific disease control	[43]

ECEA, extended cost-effectiveness analysis; GDP, gross domestic product; IRR, internal rate of return; QALY, quality-adjusted life-years; RSA, risk sharing agreements.

## Discussion

In this paper, we argue that vaccines have a broader impact beyond individual benefit and the reduction of vaccine preventable disease burden alone when compared with treatments and therefore, their economic assessment should preferentially be evaluated at the population and/or societal levels. In addition, budget impact analysis is crucial for vaccines [57]. An initial high investment is required, which will have an important pay-off depending on factors such as vaccine uptake, coverage, efficacy, and herd protection. Given those different elements, many stakeholders could be interested in understanding the economic value of vaccines and therefore the decision-making process could be more complex than for a treatment drug. Different stakeholders mean that different values should be measured and more methods of evaluation may need to be considered. Currently there is no tool available for integrating the different aspects presented here: higher level of evaluation, more value types, more stakeholders, more evaluation techniques, more decision-makers, and more different environments. Perhaps because of the lack of an integration tool, economic evaluation of vaccines appears to be moving in too many different directions, without any guidance on the most appropriate pathway to follow. This is one of the reasons we considered developing supporting frameworks, where additional vaccine benefits can be investigated more systematically and where we can be confident that the many different value aspects of a new vaccine are considered. This is discussed in the third paper of this series.

The analysis and the results presented above raise a few critical questions that need more clarification. One issue is how much effort should be made to collect precise information on disease effects that could be avoided by the vaccine but receive no medical attention, as these are often not measured. The answer will be disease- and vaccine-specific, but may be critical in demonstrating where the bulk of the benefit could make the difference between a vaccine and an existing treatment. If much of the vaccine benefit is besides the reduction of severe disease events a reduction in addition of mild and moderate disease events that could be much more frequent than the severe ones requiring medical attention, the benefit shift makes it clear that the health care system may not endorse much of the full gain of the vaccine investment. Employers and employees may gain most, potentially along with the social security programme, if they pay for persons being absent from work when taking care of family members. The latter depends on country-specific regulations, but under such circumstances the question becomes who should pay for the vaccine, when, and how [58]? Stakeholders other than the healthcare system may be interested in having the vaccine implemented as well.

Many of the other benefits of vaccines, besides the health gain, are positive outcome measures that may be difficult to demonstrate with strong evidence through simple studies. This may be because the outcome is likely to be hidden (such as improvement in QoC), or difficult to quantify (such as disease control that may evolve to elimination), or challenging to calculate precisely (such as herd protection in unvaccinated at-risk people). Such benefits could be evaluated through modelling exercises, scenario analysis, and/or retrospective data comparison. This raises the question of how easily decision-makers accept model designs with the assumptions made to support the policy direction taken. There is an open debate about the credibility, transparency, and validity of models developed and explained [59–61]. It should be part of the assessment that model constructions should be verified with observed data evaluations over time to support initial estimates of the value of vaccines.

Adjustments have been made to assessments of the economic value of vaccines using more complex models to estimate herd protection, or to design evaluation packages that go beyond the ICUA (Table 2). Do these additional evaluation tools help the decision-maker in his policy-making programme: does he get the additional and better information needed to make a good decision? Stakeholders who know about health economic evaluations want to know more, while those who do not know are not curious enough to explore new fields of economic information. This is a conclusion we came to, based on a small survey we recently conducted among decision-makers in 12 different countries in Europe [62]. There is a need and desire for more training and better education. However, we do not know enough about the internal processes of decision-making within a Ministry of Health or within a government of how a new vaccine is finally endorsed. Industry and others conduct economic assessments in the way they have been trained to do, but is that the most critical path for the decision? Cost-effectiveness analysis will help to assess the value-for-money discussion: is the right cost paid for the value of the vaccine or should we better consider the total cost of vaccination including its implementation cost? If the ICUA is the mechanism of evaluation with a fixed threshold, then the focus will be mainly on health gain. A cost-benefit analysis may allow for a broader perspective, or optimisation modelling may shift the focus to outcome measures other than health gain. However, who would be interested in that if the Ministry of Health fixes its attention and policy on the latter mainly because of comparative selection? There is a need to open the debate and widen the view of assessment of the value of prevention, particularly vaccine prevention, to a societal level where different stakeholders are concerned and different methods of economic evaluation are accepted. If we do not push for that, we fall into the trap of a narrow evaluation and no chance for change. Some health economists may argue that the new evaluation tools proposed, such as optimisation modelling, have no precise link with economic theory of welfare development through healthcare investment [63]. However, optimisation modelling can be conducted to maximise a benefit such as health gain. It makes the selection of certain options more explicit, given the constraints on budget and other limitations. In that respect it has the potential for even more transparency in selection of options and cost setting of new interventions. The matter should stay open to other approaches, improving understanding of differences between treatment and prevention, and how to decide on one versus the other if the benefits considered differ greatly between the options proposed.

## Conclusion

The economic evaluation of a new medical intervention may differ, depending on whether it is a new treatment drug, a new preventative vaccine, or a new indication for a vaccine in a new target population. Currently, the conventional approach established for treatment drugs is applied to vaccines. However, this may not be appropriate for them if the current tool does not fully capture all the benefits of vaccination. At least four elements – population rather than individual benefit, societal rather than healthcare evaluation, budget focus as well as cost-effectiveness analysis, and short-term benefit versus long-term gain – indicate that additional evaluation frameworks should be sought to improve the assessment of vaccines. So far, the focus has been on adapting the incremental cost-effectiveness ratio with adjustments to cost-evaluation (including indirect cost), to vaccine effectiveness (indirect effect), and shifting of medical offering from treatment to prevention. These changes could be helpful to stakeholders who are familiar with the method of cost-effectiveness analysis. However, vaccines have a large societal benefit and therefore involve multiple stakeholders, some of whom may see approaches other than cost-effectiveness analysis in the economic assessment of new preventative interventions. It is up to us to develop a framework to satisfy additional demands from different stakeholders.

## Highlights

- Prophylactic vaccines and therapeutic drugs are using the same economic assessment method of incremental cost utility analysis (ICUA) but it is suspected that this approach undervalues the former.
- Most often, the benefit created by new medical interventions is measured during the period of medical observation and therefore this approach may miss the benefit caused by prevention including vaccination outside this period such as the avoidance of mild diseases and chronic disease sequels. In addition, wider societal benefits can be measured with vaccines: benefit improvement through herd protection, reduction in absenteeism, better quality of care, reduction in anti-microbial resistance, a higher level of medical cost-offset, or a better disease control status that can lead to elimination.
- The ICUA method has been adapted to deal with these additional benefits using more complex models, population instead of cohort design, selecting different perspectives depending on decision maker’s needs, using optimisation algorithms, but it still may not meet all the expectancies about total value measurement seen from different angles.
- Three critical questions remain: disease events not falling under the attention of medical care but avoided by vaccination programs should only be investigated when appropriate because of the difficulties of an easy assessment; modelling and simulation approaches about suspected evidence will only be accepted by decision makers when using very transparent analysis methods; cost-benefit and optimisation modelling could be better tools for the economic assessments of vaccines instead of ICUA.

## Note

Based on our study, an oral presentation was presented at the European Society for Paediatric Infectious Diseases (2015) – 33rd Annual Meeting, 12–16 May, Leipzig, Germany; and a poster was presented at ISPOR-EU (2015) International Society for Pharmacoeconomics and Outcomes Research – 18th Annual European Congress, Milan, Italy.
