# Supplementary material for: 2. How is the economic assessment of vaccines performed today?
Source: J Mark Access Health Policy. 2017 Aug 31;5(1):1335163. doi: 10.1080/20016689.2017.1335163 (PMC5956288; doi:10.1080/20016689.2017.1335163)
Supplement: Supplemental_files.zip [file ZJMA_A_1335163_SM5630.zip › Supplemental files/Cauliflower series_transcript.docx]

Slide 1

This video summarizes the findings from 3 papers by Baudouin Standaert & Rino Rappuoli (GSK) on how the economic assessment of vaccines could be performed today. The first paper talks about the building blocks for a health economic assessment of vaccination, the second one defines how the economic assessment of vaccines is performed today, and the third one proposes a more comprehensive economic assessment of vaccines.

Slide 2

In the past, the economic evaluation technique developed for drug treatment was applied for vaccines as well. Today we discover that there are at least 28 items that differentiate vaccines from treatment. We regroup them into 3 baskets of product, people and finance. We come to the conclusion that the economic focus for vaccines should be on population, society, and budget whereas for treatment the focus is on the individual, the patient, and the cost effectiveness analysis.

Slide 3

A consequence of the different focus between drug treatment and vaccines is that the benefit should be seen from several perspectives by different stakeholders operating at the population/societal level for vaccines. It is important to include these additional perspectives into our economic evaluation. This figure shows examples of 3 types of stakeholders (payer, prescriber, population) with the specific and common value packages they like to see for vaccines: instrumental and inherent vaccine values.

Slide 4

Vaccine prevention normally does more than just avoiding cases/events that are getting medical attention. The amount of additional gain seen in places where there is no medical attention (pre- and post- medical care), can be huge. It is often missed or not reported. In the pre-medical period, the gain caused by vaccination can be important when the disease frequency is high such as for mild disease. In the post-medical intervention period, we rarely report details on the recovery time avoided with the vaccine in place, even though this can be considerable for diseases like for instance meningitis.

Slide 6

We created a cauliflower toolbox that can help identifying where and for whom which type of benefit can be measured. This is depicted graphically into 3 zones: red for the effect, blue for the subject, green for the cost. The toolbox is an attempt to be comprehensive on the evaluation of vaccines across different stakeholders and value packages. Its intent is to help identifying new values for new customers we could have been missed otherwise. By putting all these different aspects of evaluation together into one assessment, we propose to use cost-benefit analysis as an overall analysis method instead of cost-effectiveness evaluation.
