# Supplementary material for: 2. How is the economic assessment of vaccines performed today?
Source: J Mark Access Health Policy. 2017 Aug 31;5(1):1335163. doi: 10.1080/20016689.2017.1335163 (PMC5956288; doi:10.1080/20016689.2017.1335163)
Supplement: Supplemental_files.zip [file ZJMA_A_1335163_SM5630.zip › Supplemental files/HE_cauliflower_ms1_SupMat.docx]

**Supplementary File 1: What is different between vaccines and therapeutic drugs?**

The list hereunder is not exhaustive but helps understanding where differences are between the two domains of active medical prevention such as vaccination and therapeutic drugs.

| **Nbr** | **Function** | **Vaccination** | **Therapeutic drugs** |
| --- | --- | --- | --- |
| 1 | Objective | ‘Vaccination’ is about prevention. A vaccine avoids disease events that normally should occur if no vaccination is given but they may not happen anymore or at a much reduced pace. Many different types of events can be avoided with a vaccine from mild that doesn’t need medical attention to very severe cases for which it is sometimes too late to get medical support. | Therapeutic drugs reduce or alleviate suffering and pain from some-one with complaints, a patient who seeks medical advice. It can lead to cure for that individual, but only persons who seek medical advice have the chance to be treated. |
| 2 | Target Group | Vaccines prevent infectious diseases amongst healthy individuals with no complaints or specific suffering. Those people are at risk or higher risk for getting a vaccine-preventable infection. But they need to be convinced of getting the vaccine which is a challenge, not a medical one but a public health one. The number of people to reach for vaccination can be very large at start. | Treatment focusses on diseased persons with complaints. This could be a very limited group of people. |
| 3 | Environment | If one gets vaccinated it also impacts his/her direct environment by reducing the transmission risk of the infection whether that person likes it or not. There is therefore sometimes a societal duty of getting vaccinated. | The patient is the focus as he/she is the one who suffers but the consequences of his/her treatment for the direct environment is less of a concern when being treated |
| 4 | Impact | With good vaccine prevention nothing should happen after receiving it. Therefore there are difficulties in measuring the benefit of vaccination as there is nothing to be measured –for instance, one cannot ask if you feel better after being vaccinated. There is much uncertainty about the benefit to be measured when introducing a vaccine, therefore modelling is required. | The benefit must be seen quite rapidly and is easily measurable by the patient and by the doctor. |
| 5 | Safety | There is a major concern about safety of the vaccine administered because the receiver is normally healthy. One likes to see no or minimal side-effects such as intussusception with rotavirus vaccination of which the increased risk should be as minimal as possible. | The first concern with treatment is the working or the efficacy that should help alleviating the pain/the suffering or could help the cure. Some level of side-effect is acceptable if the benefit/risk is much in favour of the treatment |
| 6 | Efficacy | One expects that the vaccine should be highly efficacious (vaccine efficacy should normally be above 70% of the disease events avoided at least). | The efficacy should be good but in some disease areas like cancer is a benefit of 30% considered extremely good. |
| 7 | Reactivity | No hurt or reactivity is expected when the vaccine injection is administered | Pain with the administration is less of a concern, as ‘no pain, no cure’ is often claimed about therapy. |
| 8 | Access | One expects easy access for getting the vaccine in order to enhance uptake. | Access to some products is sometimes much regulated because of obtaining the right indication. |
| 9 | Duration | One expects long duration of activity or protection with a vaccine –not a few days, but many years if not for a life-time | The working of a therapy can be limited in time (hours, days) |
| 10 | Societal | A large societal impact can be observed as a consequence of vaccinating a large group of at risk people (indirect herd protection, improvement in quality of care, reduction in work absenteeism); the societal impact when expressed in money terms can often be larger than the disease events avoided. | The societal impact can be limited unless the recovery focus is to get full integration in society of the patient treated but much happens not always under medical attention and therefor is often not accounted for. |
| 11 | Financial | Vaccination could be financially very attractive for the healthcare system (cost offset), the individual (stay at work), the government (more tax revenue through healthy people), society because more can be done economically when many disease events are avoided. | Attractive for the industry as revenue and the prescriber who sees his patient getting better with the right selection of treatment. Treatment can sometimes be very expensive and a societal solidarity principle must be developed to make treatment affordable and accessible at large. |
| 12 | Organisation | Active prevention is needed (one needs to search for the individual in getting his vaccine and not to wait). So we need to organise ourselves at best to reach the whole population at risk (efficient organisation) and to get them vaccinated. | The approach is about to wait until the patient seeks advice by which a win-win relationship is created between the patient and the doctor if a social security system is well in place |
| 13 | Evaluation | Evaluation of the vaccine effect must happen at population and not at the individual level because of risk assessment. An individual will not be able to measure the benefit of the vaccine if nothing happens because he will not know when he has benefited of vaccine prevention. However if one looks at a group of people who all do not manifest any disease event because they have been vaccinated, the group calculation may demonstrate that the vaccine has worked and has protected. | Evaluation can happen at the individual level as it is easily measurable to reduce pain and suffering and over a short time period. |
| 14 | Budget | The budget for vaccines in healthcare often comes from a different source than therapeutic drugs, is often limited, and not easy to augment because the demand or pressure to increase is low. It is a public health pressure that is different from medical corps pressure. | The drug world is a well-established domain in healthcare with many lobbying parties involved that can create high pressure on demand. |
| 15 | Purchasing | The purchasing process of vaccines at country level is different from drugs. Many are acquired through tender processes and not through reimbursement. A tender program leads to rapid price erosion if there is fierce competition. If the winner takes it all, the business doesn’t become attractive for small companies (too high risk). So, no generic vaccines are on the market. Only a few vaccine producers exist and they are exposed too great public pressure to sell at low cost for global support. Tiered pricing strategy is therefore a must to be applied across the world which makes again the business not very attractive. | The process of getting new drugs on the market in a country is well-regulated with a standardized approach following rules of Health Technology Assessment. Only in a few countries is the tender approach applied like for vaccines (see New Zealand). |
| 16 | Investment | For the purchaser/payer is there a high initial investment that often exceeds the cost of current management of the disease when a new vaccine is introduced in the market with uncertain precise return that is unplanned, when, and where. | The number of cases and the effect can be quite precisely estimated for therapeutic drugs based on epidemiological surveys with the development of budget impact models. Clear price-volume agreements can be made between producer and payer. |
| 17 | Monitoring | One cannot easily adjust a vaccination program with boosting when the vaccine starts waning unless good disease monitoring is in place. | Overall disease monitoring is not so much a critical activity as for vaccination programs |
| 18 | Switch | One cannot easily switch from therapy to vaccine prevention because of logistic and organisational reasons. | Moving from one therapy to a next best one seems easy and obvious. |
| 19 | Focus | Vaccines have that unique ability to change focus during its product life-cycle from reduction in disease burden to control of disease, over to elimination and/or eradication. The economic value of a vaccine will therefore be different whether the product is there for reducing the burden or to control the disease. | There are limited equivalent examples for therapies that over a life-cycle switches focus as the likelihood or risk for drug resistance may increase if one moves from treatment to prevention. |
| 20 | Acceptance/Hesitancy | Getting a new vaccine accepted takes a lot of time (years) before having it on the calendar for universal mass vaccination. Individuals may become hesitant about vaccination once the disease is under control because it is not present anymore. | The process is well standardized and the timing could be quite strict before a new drug is available on the market. As the direct benefit is clearly identifiable, the patient may be more confident in accepting and receiving the treatment. |
| 21 | Market type | Bringing a new vaccine in a well-organised healthcare market versus a market in development is different. In the former one goes for substitution of therapy. In the latter one goes for priority setting of an add-on program when one needs to build up a healthcare program. | The contrast between the two worlds of developing and developed is less clear for therapy as the developing world is more struggling with infectious diseases to handle first and in which prevention takes a prominent role. |
| 22 | Third party payer | Insurance companies and employers may see much more value expressed in financial benefit going for vaccination programs rather than waiting for treatment payment. | Insurance companies essentially seek at the financial difference between therapies to select the one that is cheaper and may propose risk sharing agreements when the outcome is uncertain and the price is high. |
| 23 | At risk | Understanding well who is at-risk or higher risk in a population for getting the vaccine is most critical for identifying the total economic value of the product. One needs to evaluate the additional investment for the additional prevention benefit generated by the vaccine for a go/no go at the critical cost-effectiveness threshold level when implementing catch-up scenarios. | The focus is less on identifying the right at risk population but getting the good therapy in place that works well for the suffering person and showing good results. |
| 24 | Individual | The influence of negative information on vaccines through the anti-vaccine lobbyist will influence the behaviour at the individual level. | It is less likely that one will observe a high influencing body acting against drug therapies that will change the individual behaviour. |
| 25 | Production | The development and production of a vaccine is a very risky and laborious work that is submitted to a very rigorous and permanent quality check program which makes the process cumbersome and costly to permanently obtain a high quality product. | Drugs can be based on molecule production that is much easier to develop and to control. Less hassle to get a full quality program under development and control. The production cost is therefore much lower and the process to control is more standardised. |
